# Novel Approaches to Inhibit HIV Entry

**DOI:** 10.3390/v4020309

**Published:** 2012-02-21

**Authors:** Chukwuka A. Didigu, Robert W. Doms

**Affiliations:** Department of Microbiology, Perelman School of Medicine, University of Pennsylvania, 3610 Hamilton Walk, Philadelphia, PA 19104, USA; Email: cdidigu@mail.med.upenn.edu

**Keywords:** HIV entry, gene therapy, CCR5

## Abstract

Human Immunodeficiency Virus (HIV) entry into target cells is a multi-step process involving binding of the viral glycoprotein, Env, to its receptor CD4 and a coreceptor—either CCR5 or CXCR4. Understanding the means by which HIV enters cells has led to the identification of genetic polymorphisms, such as the 32 base-pair deletion in the *ccr5* gene (*ccr5∆32*) that confers resistance to infection in homozygous individuals, and has also resulted in the development of entry inhibitors—small molecule antagonists that block infection at the entry step. The recent demonstration of long-term control of HIV infection in a leukemic patient following a hematopoietic stem cell transplant using cells from a *ccr5∆32* homozygous donor highlights the important role of the HIV entry in maintaining an established infection and has led to a number of attempts to treat HIV infection by genetically modifying the *ccr5* gene. In this review, we describe the HIV entry process and provide an overview of the different classes of approved HIV entry inhibitors while highlighting novel genetic strategies aimed at blocking HIV infection at the level of entry.

## 1. Introduction

**Figure 1:**
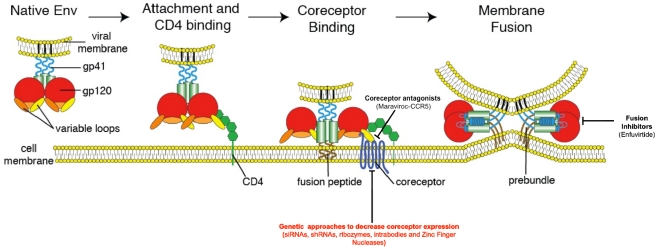
**The HIV Entry Process**. The figure below outlines a model for HIV Entry. The entry process begins with binding of gp120 (red) to its primary cellular receptor CD4 (green). CD4 binding results in conformational changes that allow binding of gp120 to the coreceptor-either CCR5 or CXCR4. Coreceptor binding results in triggering of the fusion machinery and formation of the six-helix bundle required to drive fusion of the viral and host cell membranes. Also pictured are the two main steps that have been successfully targeted (coreceptor binding and viral fusion—approved therapeutics appear in parentheses) along with the primary target of most genetic therapies aimed at preventing HIV entry—the HIV coreceptors (in red). Adapted from Antiviral Research Vol 85, Tilton J.C. and Doms R.W, “Entry inhibitors in the treatment of HIV-1 infection,” 91-100, Copyright 2009, with permission from Elsevier.

Since its identification as the etiologic agent of acquired immune deficiency syndrome (AIDS), human immunodeficiency virus (HIV) has claimed the lives of millions around the world. Although the introduction of antiretroviral therapy (ART) has dramatically altered the disease course of treated HIV-infected individuals by delaying the progression to AIDS [1,2,3], often times for many years, the considerable morbidities associated with ART (reviewed in [4]) still drive efforts to develop a cure for HIV. As one of the most intensely studied pathogens of the last decade, much is known about the life cycle of HIV, which begins with entry of the virus into susceptible cells. To enter a cell, HIV must first bind to its primary receptor CD4, and then to one of two coreceptors—CCR5 or CXCR4 (Figure 1) [5,6,7,8,9,10,11,12,13]. The choice of coreceptor used by the virus is intimately linked to disease acquisition and pathogenesis as the majority of transmitted viruses use CCR5 to enter cells [14,15,16,17], while the appearance of viruses capable of using CXCR4 during infection is associated with a more rapid progression to AIDS [18,19,20,21]. Additionally, there is a naturally occurring mutation in *ccr5* (*ccr5∆*32) that prevents expression of CCR5 on the cell surface, and individuals homozygous for this mutation are highly resistant to infection with viruses capable of using CCR5, although their cells remain susceptible to infection with CXCR4-using viruses [22,23,24]. Interest in a cure for HIV was recently reignited following the report of an HIV-infected male—the berlin patient—who developed acute myelogenous leukemia and received a hematopoietic stem cell (HSC) transplant using cells from a *ccr5*∆32 homozygous donor [25,26]. Following his transplant, he was taken off ART and his virus has remained undetectable for greater than four years, suggesting that long-term control and a possible functional cure of his disease have been achieved. This remarkable report highlights how our understanding of a very basic question—how a virus enters its host cell—has led to the development of new antiviral drugs and therapeutic approaches that have brought us a step closer to controlling the global HIV pandemic. In this review, we provide an overview of HIV entry and its impact on disease pathogenesis, and report on novel treatment approaches targeting the entry process.

## 2. HIV Entry: The Basics

Entry of HIV into target cells is mediated by the type I integral membrane viral glycoprotein Env. Env is synthesized as a polypeptide precursor termed gp160 which undergoes several of modifications within the cell as it is transported to the cell surface, including extensive N-linked glycosylation, and cleavage by cellular proteases into the extracellular gp120 and the membrane-spanning gp41 subunits [27,28,29]. The extensive glycosylation of Env contributes to its ability to evade humoral immune responses, as the carbohydrate moieties covering its surface are poorly immunogenic and may shield potentially antigenic epitopes on the glycoprotein from recognition by the immune system [30,31,32]. Following gp160 cleavage—an event required for subsequent membrane fusion—gp120 and gp41 maintain their association via non-covalent interactions and are transported to the cell surface where they exist as a trimer of heterodimers that is ultimately incorporated into the viral membrane. Prior to CD4 binding—the first essential step in HIV entry—a number of cell-surface molecules are capable of mediating Env-dependent attachment of the virion to target cells. One such attachment factor is the C-type lectin CD209 or dendritic cell-specific intercellular adhesion molecule (ICAM) grabbing non-integrin (DC-SIGN). Expressed on dendritic cells, DC-SIGN and several other lectins are capable of binding Env and boosting infection *in vitro* by facilitating *trans*-infection of surrounding CD4 T cells by dendritic cell-bound virions (reviewed in [33]). More recently, monomeric gp120 from some HIV strains has been shown to bind the gut homing integrin α4ß7 [34,35], which is expressed on activated CD4 T cells. This finding is of particular interest as the depletion of CD4 T cells in the gut-associated lymphoid tissue (GALT) early in infection is a hallmark of HIV disease [36,37,38] and ß7 integrins mediate trafficking of lymphocytes to the gut mucosa [39]. However, it is not clear whether α4ß7 supports binding of trimeric Env on the surface of virions, and thus its relevance to the gut pathology associated with HIV infection remains unknown. Further, while interactions with these and other attachment factors influence HIV infection *in vitro*, little is known regarding their significance *in vivo*.

The gp120 subunit of Env is composed of five relatively conserved (C1–C5) and five more variable (V1–V5) domains [40]. The conserved regions form the proximal core of gp120 while intrachain disulfide bonds in the variable regions of gp120 result in the formation of five variable ‘loop’ structures that make up the most exterior portion of the gp120 ectodomain [41]. The CD4 binding site on gp120 is a well conserved cavity formed at the interface of the inner and outer domains of the glycoprotein [42]. Following binding of gp120 to CD4, a series of conformational changes occur including the rearrangement of two pairs of ß-sheets from the gp120 inner and outer domains that come together to form a four-stranded ß-sheet structure termed the bridging sheet. The bridging sheet links the inner and outer domains of gp120 and interacts with the viral coreceptor, be it CCR5 or CXCR4 [42,43]. gp120 binding to CD4 also results in enhanced exposure and reorientation of the V1/V2 and V3 loops of gp120, outward rotation of each gp120 monomer to reveal the gp41 stalk, and hinge-like movements in CD4 that are thought to bring the viral membrane in close proximity to the target cell [42,44,45]. Together, all of these events culminate in the creation and exposure of the coreceptor-binding site.

The HIV coreceptors belong to the family of chemokine receptors—seven-transmembrane G-protein coupled receptors with prominent roles in immune cell trafficking. These receptors have three extracellular and intracellular loops, extracellular N-termini, and intracellular C-termini. While several different chemokine receptors are capable of mediating HIV entry *in vitro*, current evidence suggests that the CCR5 and CXCR4 chemokine receptors are the most frequently utilized *in vivo* [46,47,48]. Viruses capable of utilizing CCR5 alone, CXCR4 alone, or both coreceptors, are labeled R5-tropic, X4-tropic, and RX54 or dual-tropic viruses respectively. CCR5 is the primary coreceptor for the majority of HIV-1 isolates and is expressed on CD4+ T-cell subsets, macrophages and dendritic cells, while CXCR4 is less commonly used, but is expressed on a wide variety of cells both within and outside the immune system [49]. For reasons that remain unclear, the majority of transmitted viruses utilize CCR5 irrespective of the route of transmission and despite the availability of target cells expressing CXCR4 [17,50,51]. Multiple lines of evidence including mutational analyses, studies of small molecule inhibitors, and inhibition by coreceptor-specific blocking antibodies suggest that both the second extracellular loop (ECL2) and sulfated tyrosines within the N-terminus of the coreceptor interact with the V3 loop of gp120 and mediate coreceptor binding [52,53,54,55,56,57]. The V3 loop is also known to be a key determinant of coreceptor preference as the presence of positively charged amino acids at positions 11 and or 24/25 of V3 is correlated with CXCR4 usage [58,59].

The HIV fusion machinery is contained within the gp41 subunit of Env, which is comprised of a large cytoplasmic domain, a membrane-spanning segment, and an ectodomain that maintains contact with gp120. The ectodomain contains a typical fusion peptide—a stretch of hydrophobic amino acids at the N-terminus [60,61]—along with two α-helical heptad repeats (HR), the N-terminal HR1 and the C-terminal HR2 repeats [62,63]. The current model of gp41-mediated fusion is based on studies performed using HIV fusion inhibitors, crystal structures, and structural similarities between gp41 and other well-characterized type I membrane fusion proteins including the influenza virus glycoprotein, hemagglutinin (HA) [63,64,65,66]. In this model, the sequential interaction of Env with CD4 and a coreceptor results in exposure of the fusion peptide, which then inserts into the plasma membrane of the host cell, causing gp41 to physically link both membranes. Subsequently, the three HR1 domains of the Env trimer interact with one another to form a coiled coil, and the three HR2 segments fold back on the HR1 trimer creating a six-helix bundle that brings the viral and host cell membranes in close contact with one another, allowing for mixing of the two membranes and formation of the fusion pore. Fusion between Env and the host cell was long thought to occur at the plasma membrane as HIV entry occurs in a pH-independent manner [67] and Env is capable of mediating fusion between neighboring cells, provided that they express CD4 and an appropriate coreceptor [68]. However, recent work using *trans* dominant-negative mutants of proteins involved in clathrin-mediated endocytosis [69] along with elegant studies using single-virion imaging [70] have demonstrated a clear role for components of the endocytic pathway in HIV entry in a number of cell lines. These studies were performed using immortalized cell lines, and as such, the role of the endocytic pathway in HIV entry into relevant cell types *in vivo* will need to be determined. 

## 3. Inhibition of HIV Entry

The multi-step process by which HIV enters cells provides a series of unique targets for interventions to prevent viral entry including receptor and coreceptor binding, and membrane fusion. Efforts to inhibit these steps have led to the discovery of a new class of anti-HIV drugs—the HIV entry inhibitors (reviewed in [71]). A number of CCR5 inhibitors have been developed and display anti-HIV activity both *in vitro* and *in vivo*. These drugs are believed to work by binding to CCR5 at a site distinct from the gp120-binding site and subsequently alter the conformation of the CCR5 extracellular loops required for entry of R5-tropic HIV variants. One such CCR5 antagonist, *maraviroc*, is licensed for use in the United States and in Europe [43,72]. A variety of CXCR4 antagonists have also been developed, and while they exhibit potent anti-HIV activity *in vitro* (against X4 but not R5 virus strains), administration of these drugs *in vivo* results in mobilization of HSCs from the bone marrow to the peripheral blood, highlighting the important role of CXCR4 in HSC homing [73,74,75]. Although this side effect limits their use in HIV-infected individuals, the CXCR4 antagonist *plerixafor*, is currently used to mobilize HSCs for subsequent autologous transfer in patients with non-Hodgkin’s lymphoma and multiple myeloma [76]. 

gp41-mediated membrane fusion presents another drug target in the HIV entry process. Synthetic peptides based on the sequence of HR2 display significant antiviral activity against HIV *in vitro* but for many years, the mechanism of this antiviral activity remained unknown [77]. However, both the observation that these peptides display higher antiviral activity as dimers, and the elucidation of the structure of the gp41 fusion machinery have led to a model for their mechanism of action [63,64,78]. These drugs are now believed to act in a dominant negative fashion by competing with the HR1 and HR2 domains of gp41 and ultimately preventing the formation of the six-helix bundle required for membrane fusion. One such peptide, *enfuvirtide*, is the only FDA-approved HIV fusion inhibitor, and is indicated for use in combination with standard antiretroviral therapy. However, the twice-daily subcutaneous dosing schedule of the drug makes it an unattractive choice for many patients and care providers. 

As is the case for most anti-HIV drugs, viral variants resistant to all of the entry inhibitors have been identified. The appearance of *maraviroc* resistant viruses *in vitro* is a well-established phenomenon and these viruses either adapt to recognize the drug-bound conformation of CCR5, or more commonly, acquire the ability to use CXCR4 in addition to CCR5 (reviewed in [79]). In vivo, resistance to *maraviroc* most commonly results from outgrowth of pre-existing X4 or R5X4 variants that are sometimes present at very low levels prior to the onset of therapy. As such, tropism testing is indicated prior to administration of *maraviroc*. In the case of *enfuvirtide*, mutations within HR1 and HR2 rapidly select for viruses with a dramatically decreased sensitivity to the drug [80,81]. HIV drug resistance is not unique to entry inhibitors, but is a widespread problem seen with all classes of HIV chemotherapeutics, and while ART increases survival in HIV-infected individuals, the problems of drug resistance, impaired immune function despite ART, long-term financial cost and drug-associated toxicities of ART continue to fuel the search for curative therapies for HIV infection. Although the search for a cure has long been met with an aura of skepticism, the success of the berlin patient has provided hope that a cure is indeed achievable. After living with HIV for at least 10 years, and experiencing control of viremia on ART for 4 years, the berlin patient developed acute myelogenous leukemia and received a HSC transplant using cells from a *ccr5*∆32 homozygous donor. His ART was stopped 1 day before the transplant and despite being able to detect proviral HIV DNA for up to 60 days post-transplant, he never experienced a rebound of his viral load [26]. In the 4 years that have followed this transplant, his viral load has remained undetectable as determined by viral RNA and DNA assays of multiple tissues including the gut, bone marrow, peripheral blood and brain. He has also experienced a repopulation of his gut mucosa CD4+ T cells along with a decrease in antibodies directed against HIV. 

The Berlin patient received two interventions—conditioning chemotherapy and radiation, and an infusion of HIV resistant cells—all of which of which could have contributed to his dramatic control of the infection. However, as similar chemotherapy regimens and HSC transplants using cells from normal donors have always been followed by a recrudescence of infection following cessation of ART (reviewed in [82]), the control of infection seen in the Berlin patient was clearly dependent upon the genetic absence of CCR5, reinforcing the important role of HIV entry in the maintenance of an established infection. Despite the urgent need for a cure for HIV infection, the risks associated with chemotherapy and radiation, and the relatively low frequency of *ccr5∆*32 homozygous individuals, makes it unlikely that allogeneic HSC transplants using cells from *ccr5∆*32 homozygous donors will become a widespread treatment option, and has prompted attempts to mimic the genetic knockout of CCR5 that was achieved in the Berlin patient. 

## 4. Genetic Knockout of CCR5

Several studies have successfully decreased expression of CCR5 in primary human HSCs, CD4 T cells and macrophages *in vitro* using a variety of unique genetic approaches. Dominant negative forms of CCR5, anti-CCR5 intracellular antibodies (intrabodies), and a number of RNA-based approaches including RNA interference (RNAi), short-hairpin RNAs (shRNA), and ribozymes have been all been shown to decrease CCR5 expression [83,84,85,86,87,88]. In one recent study, the investigators took a combinatorial approach by simultaneously targeting CCR5, the viral genes *tat* and *rev*, and the viral trans-activating region (TAR) [83]. Using this approach, the authors successfully introduced an shRNA construct targeting a *tat/rev* exon, a TAR decoy that localized to the nucleolus, and a CCR5-targeting ribozyme into human HSCs *ex vivo* using a lentiviral vector, and showed that the cells expressing the construct—dubbed Triple-R—were protected from infection *in vitro* and provided protection from infection in a humanized mouse model of HIV infection. Following this promising pre-clinical study, Triple-R was tested in a Phase I trial where four patients with AIDS-related lymphoma who were scheduled to receive autologous HSC transplants had a fraction of their infused stem cells modified by the Triple-R construct [89]. The gene-modified cells engrafted in all four patients, and were readily detectable at a consistently low level for up to 24 months. While these cells did not have any detectable effect on infection, possibly due to the low number of gene-modified cells, the study was successfully completed without any toxicity related to the genetic modification of HSCs suggesting that modification of these long-term progenitor cells is a safe and viable option for future genetic therapies aimed at eliminating HIV infection. 

**Figure 2:**
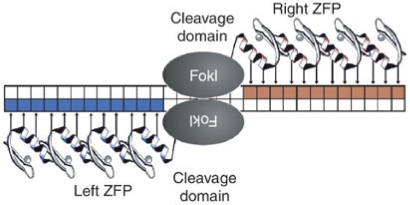
Zinc Finger Nucleases (ZFNs) bind and cleave DNA. A ZFN consists of a DNA-binding zinc finger protein (ZFP) domain fused to the catalytic domain of a *Fok*I endonuclease. Each ZFP array in the image (ZFP left & right) contacts 12bp of DNA for a total DNA specificity of 24 base pairs. The members of the ZFN pair depicted are separated by a 5 to 6 base pair spacer and concomitant binding of each ZFN results in dimerization of the nuclease domains and the introduction of a double stranded break. Adapted by permission from Macmillian Publishers Ltd: Nature Biotechnology. Miller, J.C.; Holmes, M.C.; Wang, J.; Guschin, D.Y.; Lee, Y.-L.; Rupniewski, I.; Beausejour, C.M.; Waite, A.J.; Wang, N.S.; Kim, K.A.; *et al*. An improved zinc-finger nuclease architecture for highly specific genome editing. *Nature biotechnology* **2007**, *25*, 778-785. Copyright 2007.

As with most gene therapy approaches, achieving lasting expression of the transgene of interest in progenitor cell populations such as HSCs is a challenge, and there remains a need for the development of vector systems that will allow for stable transgene expression in different cell populations over long periods of time. A limitation of a number of these studies has been the need for repeated administration of many of these agents due to their transient effects. As such, recent work has sought to introduce somatic mutations into the *ccr5* gene with the ultimate goal of phenocopying the natural *ccr5∆32* mutation by rendering a fraction of cells *ccr5* negative. These mutations were introduced using zinc finger nucleases (ZFNs)—chimeric proteins composed of a DNA-binding zinc-finger protein (ZFP) fused to the catalytic domain of a *FokI* restriction endonuclease (Figure 2) [90]. Following binding of a ZFN pair to the intended target sequence, the nuclease domains of the ZFN pair dimerize and introduce a double stranded break (DSB) in the DNA molecule. This break is repaired by the error-prone cellular non-homologous end joining (NHEJ) machinery and typically results in the introduction of insertions and deletions that render the gene product non-functional [91,92,93]. As the ZFP domain of a ZFN can be engineered to bind unique DNA sequences, a pair of ZFNs that recognize the portion of the *ccr5* gene encoding the first transmembrane segment were designed and shown to specifically modify 20–40% of the CCR5 alleles in human CD4 T cells *in vitro* [94]. These gene-modified cells appeared indistinguishable from wildtype cells in culture, but following challenge with an R5-tropic virus, the gene-modified cells had a selective growth advantage as they were protected from infection. *In vivo* experiments using a humanized mouse model of HIV infection also showed that CCR5-modified CD4 T cells engrafted into the animals at levels similar to unmodified CD4 cells, and are capable of controlling viral load and maintaining CD4 counts following infection with R5-tropic viruses. More recently, CCR5-specific ZFNs have been shown to be capable of disrupting the CCR5 gene in human HSCs *ex vivo*, with CCR5-modified HSCs displaying normal multi-lineage differentiation into a variety of CCR5-negative hematopoietic cell subsets that recapitulate the protective phenotype seen in the *in vitro* and humanized mouse studies using CCR5-modified CD4 T cells [95]. The ultimate goal of ZFN therapy is the *ex vivo* modification of CD4 T cells and HSCs from HIV infected individuals, with the hope that after reinfusion, these cells will control HIV infection in the absence of ART. To this end, the safety and tolerability of CCR5-ZFN modified CD4 T cells is currently being tested in two ongoing human clinical trials (NCT00842634, NCT01044654; Clinicaltrials.gov) and preliminary results presented at the 2011 Conference on Retroviruses and Opportunistic Infections (CROI) suggest that infusion of ZFN-modified CD4 T cells into patients currently on ART is safe, and these gene modified cells appear to initially expand and persist at a stable level over time. While these human studies provide evidence for the safety of this approach and its ability to be scaled up, the effect of infusing CCR5-ZFN modified cells on HIV disease can only be assessed in the absence of ART. As such, structured treatment interruptions, where patients are taken off ART following infusions of CCR5-ZFN modified cells, have been built into the human trials. One of the benefits of the ZFN approach is the permanent modification of the *ccr5* gene in a subset of cells, thereby getting around the problem of longevity that is intrinsic to approaches targeting CCR5 at the level of RNA. Additionally, the *ex vivo* modification step bypasses the need for systemic *in vivo* delivery of the gene therapy vector and thus potentially limits toxicity while ensuring that only the intended cells receive the CCR5 ZFN.

## 5. Future Directions

Despite their promise, genetic approaches targeting CCR5 are currently incapable of achieving the complete genetic knockout of CCR5 that exists in *ccr5∆32* homozygous individuals and was accomplished in the Berlin patient. Whether the incomplete ablation of CCR5 provided by these genetic approaches will be able to provide long-term, or indeed, any control of established HIV infection remains to be seen. Additionally, it is unclear how the virus will respond to the partial ablation of one of its primary coreceptors. One possible outcome is a switch from R5 to X4-tropism or an outgrowth of a pre-existing X4-tropic virus reservoir following removal of *ccr5*. As the coreceptor switch correlates with an accelerated disease progression, a possible coreceptor switch following the removal of *ccr5* may be minimized by performing tropism testing to exclude patients with detectable levels of X4-tropic virus prior to administering treatments that reduce levels of CCR5 expression, or by the use of approaches that simultaneously target both coreceptors [96]. However, this latter goal may prove challenging because of the important role of CXCR4 in immune cell trafficking. Additionally, HIV is capable of using a number of other chemokine receptors *in vitro,* and recent reports have identified variants of the closely related simian immunodeficiency virus (SIV_smm_) that utilizes CCR5 but not CXCR4 *in vitro*, yet still infects sooty mangabeys lacking functional CCR5—suggesting *in vivo* usage of coreceptors other than CCR5 and CXCR4 [97]. This raises the possibility that another potential mechanism of escape from therapies involving the genetic ablation of the major coreceptors is viral evolution to use some of the less commonly utilized alternative coreceptors. Although a number of questions remain regarding the feasibility, efficacy, and long-term toxicities of these approaches to genetically modify the HIV coreceptors, as well as their effects on viral evolution, genetic ablation of the entry factor CCR5 remains the only proven means of providing long-term control of infection in the absence of ART and as such, continues to provides hope that a cure for HIV can be achieved.
